# EC2KEGG: a command line tool for comparison of metabolic pathways

**DOI:** 10.1186/1751-0473-9-19

**Published:** 2014-09-02

**Authors:** Aleksey Porollo

**Affiliations:** 1 Center for Autoimmune Genomics and Etiology, Division of Biomedical Informatics, Cincinnati Children’s Hospital Medical Center, 3333 Burnet Avenue, Cincinnati, OH 45229, USA

## Abstract

**Background:**

Next-generation sequencing and metagenome projects yield a large number of new genomes that need further annotations, such as identification of enzymes and metabolic pathways, or analysis of metabolic strategies of newly sequenced species in comparison to known organisms. While methods for enzyme identification are available, development of the command line tools for high-throughput comparative analysis and visualization of identified enzymes is lagging.

**Methods:**

A set of perl scripts has been developed to perform automated data retrieval from the KEGG database using its new REST program application interface. Enrichment or depletion in metabolic pathways is evaluated using the two-tailed Fisher exact test followed by Benjamini and Hochberg correction.

**Results:**

Comparative analysis of a given set of enzymes with a specified reference organism includes mapping to known metabolic pathways, finding shared and unique enzymes, generating links to visualize maps at KEGG Pathway, computing enrichment of the pathways, listing the non-mapped enzymes.

**Conclusions:**

EC2KEGG provides a platform independent toolkit for automated comparison of identified sets of enzymes from newly sequenced organisms against annotated reference genomes. The tool can be used both for manual annotations of individual species and for high-throughput annotations as part of a computational pipeline. The tool is publicly available at http://sourceforge.net/projects/ec2kegg/.

## Background

Next-generation sequencing enabled large scale projects, including metagenomics and metatranscriptomics. There is a growing need for computational high-throughput pipelines that would facilitate the genome- or transcriptome-wide annotations of identified genes and their subsequent comparative analysis with other annotated organisms. The new posed questions may include: how do metabolic strategies differ between a free living organism and its taxonomically close congener, which is an obligate parasite? What strategies do bacteria employ in their metabolism to adapt to different niches of a host, e.g. in blood *versus* gastrointestinal microenvironment?

Annotation of individual genomes with respect to identification of enzymes has been well developed and implemented in various packages, such as PRIAM [1], SHARKhunt [2], Blast2GO [3]. However, these tools do not provide comparative analysis of metabolic pathways between different organisms with subsequent visualization of results. This limitation has been addressed by some approaches, such as Comparative Pathway Analyzer [4] or ComPath [5]. Unfortunately, most of these web-servers are no longer maintained nor operational, with no stand-alone versions provided. The most up-to-date and fully operational web-server currently available to achieve these tasks is KEGG Mapper (http://www.kegg.jp/kegg/mapper.html) [6], but it requires conversion of EC numbers to KEGG ontology identifiers and does not provide statistics about overlapping enzymes and pathways. On the other hand, DAVID [7] performs the pathway enrichment analysis with subsequent visualization of these pathways. However, the enrichment statistics considers the pathways as a whole, indivisible set of enzymes without accounting for the fact that pathways may consist of functional modules, e.g. as defined in the KEGG Module database. Hence, a given list of genes and a reference genome may contain the same number of non-overlapping enzymes within a pathway, and this will not be reflected in the enrichment tests.

EC2KEGG has been developed to provide a command line tool for the automated comparative analysis of metabolic pathways between two organisms. In addition to the pathway enrichment analysis, the tool produces report about both the shared and unique enzymes for each organism, generates URL strings to visualize data using the KEGG Pathway maps. It also lists enzymes that are not part of the defined metabolic pathways.

## Methods

### Input

A query list of enzymes represented by EC numbers has to be obtained using a third party software, see e.g. the Background section. Information about organism specific genes, enzymes, and pathways is automatically retrieved from the KEGG database using its new representational state transfer application programming interface (REST API). Definitions of annotated organisms, pathways and their corresponding lists of enzymes are included in the package. However, it is recommended to periodically execute the enclosed get_definitions.pl script to update the definitions used by EC2KEGG.

### Statistics

Since a given pathway can be over- or under-represented, the two-tailed Fisher exact test is employed to test whether a pathway is significantly differently represented in a given list compared to a reference genome. The background is defined by the entire list of genes in a reference genome. The number of genes in a pathway is defined by KEGG annotation for a given reference organism. P-values are subsequently adjusted to the multiple hypotheses testing using Benjamini and Hochberg correction [8]. Of note, the employed perl statistical module has other commonly used corrections implemented, and the main perl script (ec2kegg.pl) can be easily modified to change the correction method.

### Output

The report contains KEGG Pathway ID, pathway name and category, the total numbers of: (i) all genes known in a given pathway, (ii) enzymes from a reference genome belonging to this pathway, (iii) enzymes found in a given list, (iv) shared and unique enzymes. These counts are followed by the corresponding lists of EC numbers, p-values, adjusted p-values, and the URLs to visualize a KEGG pathway using the following color code: green – an enzyme unique to a reference organism, red – an enzyme unique to a given list, yellow – a shared enzyme. Output is generated in the tabulated text format that can be redirected to a file or fed to subsequent processing through a pipeline. Only those pathways are reported that contain at least one enzyme from a given list or a reference organism.

### Dependencies and restrictions

The following perl modules are required to run the EC2KEGG package: libwww-perl - for internet communication with KEGG; Text-NSP - for computing the Fisher exact test; Statistics-Multtest - for correcting p-values on multiple hypotheses testing. All these modules are freely available from the Comprehensive Perl Archive Network (CPAN, http://search.cpan.org/).

There are restrictions imposed by the KEGG database on using REST API (http://www.kegg.jp/kegg/rest/): “KEGG API is provided for academic use by academic users belonging to academic institutions. This service should not be used for bulk data downloads”. The latter restriction does not apply to EC2KEGG, as it downloads very limited information for a given request. However, for the high-throughput analyses, the user may need to obtain the license from KEGG owners.

## Results and discussion

### Package installation

The package can be downloaded from SourceForge (http://sourceforge.net/projects/ec2kegg/) and installed on a computer by unpacking the compressed file. The package consists of the definitions files (lists or organisms, pathways, and enzymes), two perl scripts, a README file, and the list of enzymes from *Saccharomyces cerevisiae* to serve as an example of a query list. EC2KEGG is platform independent and can be executed under any operating system, provided perl and three required perl modules are installed.

### Command line tools

EC2KEGG is aimed to be part of a computational pipeline and purposely has no graphic user interface (GUI). The package contains two perl scripts to be run from a command line. The first script has no parameters and is aimed to update the definitions necessary for execution of the main perl script.

>perl get_definitions.pl

The main script takes two required parameters: (i) a KEGG code for a reference organism and (ii) a file name with the list of enzymes (one per line, in the first column if the input file has multiple columns). For example, one can use the following command to compare the list of *Saccharomyces cerevisiae* (*S. cerevisiae*) enzymes against *Schizosaccharomyces pombe* (*S. pombe*):

>perl ec2kegg.pl spo sce.ec > sce2spo.txt

The list of annotated species and their KEGG codes can be found in the definitions/kegg_org.txt file enclosed in the package.

### Case study

*Pneumocystis carinii* (*P. carinii*) is a pathogenic fungus that belongs to the *Pneumocystis* genus causing Pneumocystis pneumonia in mammalian hosts with weakened immune system. Despite decades of research on the fungus, its biology remains elusive. Both genetic studies and drug development are impeded by the lack of *ex vivo* culture of this obligate organism. In the efforts of deciphering its metabolic strategies to thrive in host lungs, the genome of *P. carinii* has been sequenced and currently available at http://pgp.cchmc.org/.

Genomic sequences of *P. carinii* have been searched through for enzymes using SHARKhunt. EC2KEGG has been subsequently used to map the identified enzymes into metabolic pathways, and to compare with phylogenetically close but free living fungi: *S. pombe, S. cerevisiae*, and *Aspergillus fumigatus* (*A. fumigatus*). Table 1 contains the summary of mapping *P. carinii* enzymes to metabolic pathways and their comparison to the three reference fungi. There have been 481 enzymes identified in DNA sequences of *P. carinii*, including 328 mapped to KEGG Pathways and 153 non-mapped. *P. carinii* shows the largest overlap of enzymes with *A. fumigatus*, having shared 271 mapped and 84 non-mapped enzymes, respectively.

**Table 1:** ***P. carinii *** **enzymes annotated by EC2KEGG**

**Enzymes**	***S. pombe***	***S. cerevisiae***	***A. fumigatus***
**Mapped to pathways**			
Shared	246	261	271
Unique to reference	230	258	382
Unique to *P. carinii*	82	67	57
**Non-mapped**			
Shared	78	83	84
Unique to reference	75	96	147
Unique to *P. carinii*	75	70	69

The pathogen displays depletion of pathways in the amino acid metabolism category (Table 2), which is consistent with the recently published independent analysis of the human infecting species *Pneumocystis jirovecii*[9]. Moreover, the fungus contains the incomplete steroid biosynthesis pathway, missing Erg2, Erg3, and Erg5 enzymes downstream of the pathway. This indicates that it cannot synthesize ergosterol from precursors and has to scavenge cholesterol from its host, which has been previously pointed out in other studies [10,11], and may explain the fact that the pathogen is not susceptible to antifungal drugs targeting this pathway, such as azole-based therapeutics. The detailed per pathway information derived using EC2KEGG with entailed highlighted KEGG Pathway maps can be found at http://pgp.cchmc.org/.

**Table 2:** **Representation of amino acid metabolism by *P. carinii *** **enzymes in comparison to free living fungi**

**Pathway ID**	**Pathway**	**Pc**	**Spo**	**Pc∈**	**Pc∉**	**Sce**	**Pc∈**	**Pc∉**	**Afm**	**Pc∈**	**Pc∉**
				**Spo**	**Spo**		**Sce**	**Sce**		**Afm**	**Afm**
00250	Alanine, aspartate and glutamate metabolism	11	21	10	1	23	10	1	24	10	1
00260	Glycine, serine and threonine metabolism	10	24	7	3	27	9	1	34	9	1
00270	Cysteine and methionine metabolism	10	26	7	3	30	8	2	31	10	0
00280	Valine, leucine and isoleucine degradation	5	7	4	1	8	4	1	19	4	1
00290	Valine, leucine and isoleucine biosynthesis	0	7	0	0	8	0	0	8	0	0
00300	Lysine biosynthesis	1	10	0	1	10	0	1	11	0	1
00310	Lysine degradation	4	9	4	0	9	4	0	11	4	0
00330	Arginine and proline metabolism	8	28	4	4	28	5	3	35	5	3
00340	Histidine metabolism	1	9	0	1	10	0	1	12	0	1
00350	Tyrosine metabolism	1	9	1	0	8	1	0	18	1	0
00360	Phenylalanine metabolism	2	7	2	0	7	2	0	14	2	0
00380	Tryptophan metabolism	8	7	3	5	13	8	0	19	7	1
00400	Phenylalanine, tyrosine and tryptophan biosynthesis	10	19	10	0	20	10	0	20	10	0

Reference fungi include *S. pombe* (Spo), *S. cerevisiae* (Sce), and *A. fumigatus* (Afm).

## Conclusions

The new command line tool, EC2KEGG, enables the automated and high throughput comparative analysis of a given list of enzymes against a reference organism. In addition to computing statistically differently represented metabolic pathways, the tool produces the lists of overlapped and organism-specific enzymes, provides links to visualize highlighted maps from KEGG Pathway. Analysis of computationally identified enzymes within an under-investigated species, *Pneumocystis carinii*, indicated a number of depleted metabolic pathways compared to its free living congeners, thus providing a basis for developing testable hypotheses about nutrients missing in the media to culture the pathogen.

## Competing interests

The author declares that he has no competing interests.

## Authors**’** contributions

The perl code for EC2KEGG package and text of the manuscript were written by AP.
